# Cystatin C Is an Important Biomarker for Cardiovascular Autonomic Dysfunction in Chinese Type 2 Diabetic Patients

**DOI:** 10.1155/2019/1706964

**Published:** 2019-03-18

**Authors:** Xubin Yang, Qiongyan Lin, Xiaoshan Li, Lin Wu, Wen Xu, Yanhua Zhu, Hongrong Deng, Yao Zhang, Bin Yao

**Affiliations:** ^1^Department of Endocrinology and Metabolism, Guangdong Provincial Key Laboratory of Diabetology, The Third Affiliated Hospital of Sun Yat-sen University, No. 600 Tianhe Road, Guangzhou, Guangdong 510630, China; ^2^Department of Ultrasonography, Traditional Chinese Medicine Hospital of Yuexiu District, Guangzhou, Guangdong, China; ^3^Department of Cardiovasology, The Third Affiliated Hospital of Sun Yat-sen University, No. 600 Tianhe Road, Guangzhou, Guangdong 510630, China

## Abstract

**Background:**

Cardiovascular autonomic dysfunction is closely related to increased mortality in patients with diabetes. Previous studies have proved that cystatin C (CysC) is an important predictor of both peripheral neuropathy and cardiovascular events. However, whether CysC is also associated with cardiovascular autonomic dysfunction remains unclear. Therefore, the aim of this study was to investigate the relationship between CysC and cardiovascular autonomic dysfunction in type 2 diabetic patients without renal dysfunction.

**Methods:**

A total of 161 type 2 diabetic patients with normal serum creatinine (less than 133 *μ*mol/l) and estimated glomerular filtration rate (eGFR) higher than 60 ml/min per 1.73 m^2^ were recruited in our study. Cardiovascular autonomic dysfunction was determined by heart rate variability (HRV) measured by a 24-hour Holter monitor. Serum CysC was tested by particle-enhanced turbidimetric immunoassay, and subjects were divided into three groups based on the tertiles of CysC. Pearson correlation analysis was used to evaluate the association between different indexes, and the association of CysC with HRV indexes was assessed by multivariate linear regression analysis.

**Results:**

The HRV parameters were lower in the group with the highest CysC concentration than in the groups with lower levels of CysC (*P* < 0.05). Pearson correlation analysis showed a negative relationship between CysC and the HRV parameters, including SDNN (*r* = −0.31, *P* < 0.001), SDANN (*r* = −0.25, *P* = 0.002), and logLF (*r* = −0.18, *P* = 0.023). Furthermore, multivariate linear regression analysis revealed that CysC was independently correlated with SDNN (*β* = −24.11, *P* = 0.015) and SDANN (*β* = −19.88, *P* = 0.047) after adjusting for the confounding factors of gender, age, blood pressure, body mass index, eGFR, and hemoglobin A1c.

**Conclusions:**

Serum CysC levels are associated with cardiovascular autonomic dysfunction; furthermore, CysC may be a reliable and convenient biomarker for detecting cardiovascular autonomic dysfunction.

## 1. Introduction

Cardiovascular autonomic neuropathy, the impairment of cardiovascular autonomic nerve function, is one of the most important but overlooked complications of diabetes for its association with cardiovascular morbidity and mortality but mostly asymptomatic in patients with diabetes [1–3]. Previous studies have proved that strictly controlling risk factors of cardiovascular autonomic dysfunction can delay the progress of impaired cardiovascular autonomic nerve function or even reverse its nature course [4]. As a result, it is essential to identify cardiovascular autonomic dysfunction at the early stage. In previous studies, it has been reported that cardiovascular autonomic dysfunction could be detected early by reduced HRV [5, 6]. However, the requirement of specialized personnel of HRV test limits its use in general practice, which eventually leads to the failure to decrease overall prevalence and progression of cardiovascular autonomic dysfunction [7]. Thus, it is necessary to explore a more accessible index to identify cardiovascular autonomic dysfunction in diabetes, especially at early stages when prevention is most available.

Cystatin C (CysC), which is generated by nucleated cells and removed from the bloodstream by the glomeruli, has been considered a biomarker of the glomerular filtration rate that is superior to creatinine and the estimated glomerular filtration rate (eGFR) [8, 9]. In a previous study, it was reported that the concentrations of CysC were stable and not influenced by extrarenal factors such as sex, age, and muscle mass [10]. Recently, several studies have revealed that a higher level of serum CysC was independently associated with an increased risk for cardiovascular events, even in individuals without existing chronic kidney disease diagnosed by eGFR values and creatinine levels [11, 12]. Furthermore, several studies have also reported that CysC may be a marker of both central and peripheral nervous system diseases [13, 14]. Therefore, in addition to its role as an indicator of impaired renal function, CysC may play a key role in the early detection of cardiovascular disease and peripheral neuropathy. However, the relationship between CysC and the impairment of cardiovascular autonomic function in diabetes has not yet been fully elucidated.

Therefore, this study was conducted to explore the relationship between cardiovascular autonomic dysfunction and CysC in patients with type 2 diabetes without renal dysfunction.

## 2. Materials and Methods

### 2.1. Study Population

In total, 161 type 2 diabetic patients who were hospitalized mostly for the poor control of blood glucose levels in our hospital were enrolled from July 2013 to June 2014. Clinical data, including gender, age, height, weight, and diabetes duration, were obtained for all patients by a well-trained researcher. Serum CysC, blood lipid profiles, hemoglobin A1c (HbA1C), and other health-related variables were measured. Patients were excluded by the following conditions: (1) arrhythmia, coronary heart disease, and heart failure; (2) taking beta-blockers or glucocorticoid for the past 2 weeks; (3) history of cerebral infarction; (4) severe infection, respiratory illness, or hematologic disease; (5) acute complications of diabetes, including diabetic ketosis acidosis, diabetic hyperosmolar coma, and severe foot ulcer; and (6) serum creatinine higher than 133 *μ*mol/l and estimated glomerular filtration rate (eGFR) < 60 ml/min per 1.73 m^2^ which was considered renal dysfunction [15]. This study was approved by the ethics committee of the Third Affiliated Hospital of Sun Yat-sen University. Written informed consent was obtained from all participants before participation.

### 2.2. Measurements

The measurements of height and weight, based on a standardized form, were recorded by the same physician. Body mass index (BMI) was calculated as body weight (kg) divided by the square of the height (m). The blood pressure level was calculated by the mean of two consecutive blood pressure values 10 minutes apart taken in a sitting position. Blood and urine samples were collected after a 10-hour overnight fast. Serum concentrations of total cholesterol (TC), triglycerides (TG), LDL-cholesterol (LDL-c), HDL-cholesterol (HDL-c), fasting plasma glucose, and creatinine (Cr) were obtained via an automated enzymatic method (Hitachi, Japan, 7600-020 autonomic analyzer), and high-pressure liquid chromatography (Bio-Rad, USA, D-10 analyzer) was used to evaluate the HbA1c level. The eGFR was estimated by the simplified MDRD equation [16]. The concentration of serum CysC was measured by particle-enhanced turbidimetric immunoassay (Hitachi, Japan, 7600-020 autonomic analyzer).

### 2.3. HRV Analysis

A 24-hour HRV analysis was conducted using 24-hour ambulatory electrocardiograph monitoring (Marquette, USA) of all subjects. The indexes of HRV included time domain methods: (1) the mean of the 5-minute standard deviations of NN intervals calculated over 24 hours (SDNN, ms), (2) the standard deviation of the average NN intervals calculated over 5 minutes (SDANN, ms), (3) the percentage of the interval differences of successive NN intervals greater than 50 ms (PNN50, %), and (4) the square root of the mean squared differences of successive NN intervals (RMSSD, ms). The following frequency domain methods were also computed: (1) power in the low-frequency range (LF, ms^2^), (2) power in the high-frequency range (HF, ms^2^), and (3) and low-frequency/high-frequency ratio (LF/HF) [17]. A reduction in the parameters of HRV may reflect an injury to cardiovascular autonomic nerve function: the lower the levels of HRV indexes, the more severe the injury to cardiovascular autonomic nerve function [17]. Before the tests, coffee, wine, tea, and cigarettes were prohibited for at least 24 hours.

### 2.4. Statistical Analyses

SPSS 22.0 software (SPSS Inc.) was used to perform the statistical analyses. Clinical characteristics are presented as mean ± SD for continuous variables and as frequency percentages for categorical variables. Differences between groups were evaluated by a *t*-test, a chi-square test, or the Wilcoxon rank-sum test. Data in nonnormal distributions were logarithmically transformed before statistical analysis. The subjects were categorized based on tertile cutoff points of the serum CysC levels, which were calculated at the 33rd and 67th percentiles as follows: tertile 1, CysC < 0.78 mg/l; tertile 2, 0.78-0.99 mg/l; and tertile 3, CysC > 0.99 mg/l. The relationships between different clinical parameters were assessed by Pearson correlation analysis. Variables that were significantly related to the objective variable were tested for independence using multivariate linear regression analysis. A *P* value <0.05 showed significant differences.

## 3. Results

**Figure 1:**
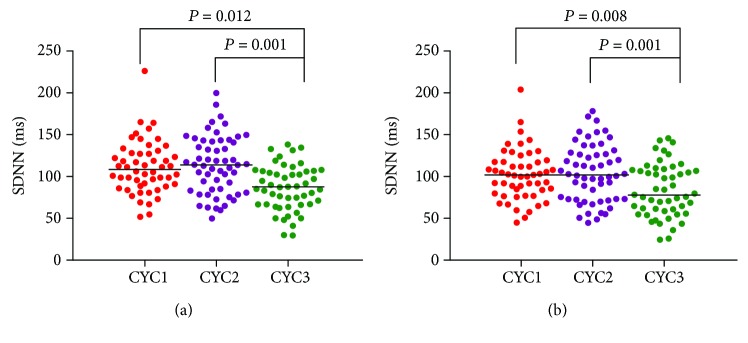
Determination of the HRV indexes in patients with different levels of CysC. (a) Determination of SDNN values in patients with different levels of CysC yielded a statistically significant trend when SDNN values were compared. Means ± SD of SDNN values for CysC 1: 110.10 ± 30.6 ms; CysC 2: 114.02 ± 34.7 ms; and CysC 3: 86.37 ± 26.8 ms. (b) Determination of SDANN values in patients with different levels of CysC yielded a statistically significant trend when SDANN values were compared. Means ± SD SDANN values for CysC 1: 100.75 ± 28.9 ms; CysC 2: 104.32 ± 34.3 ms; and CysC 3: 83.76 ± 31.5 ms.

The clinical characteristics of the subjects are presented in Table 1. Compared with patients in the group with lower levels of CysC, subjects in the groups with higher CysC concentrations were older, had longer durations of diabetes, and had higher values of creatinine (Cr), uric acid (UA), and *β*2-microglobulin (*β*2-MG). In addition, the eGFR and the frequency domain HRV variables (logLF and logHF) decreased with increasing CysC values. Moreover, the indexes of HRV, including SDNN and SDANN, were lower in the patients in CysC tertile 3 than in those in CysC tertiles 1 and 2 (Figure 1). However, there was no significant difference in HRV indexes between subjects in tertiles 1 and 2 (*P* > 0.05). Furthermore, considering age, we found that patients older than 60 years had higher CysC levels and lower SDNN indices than subjects younger than 60 years (Table 2).

**Figure 2:**
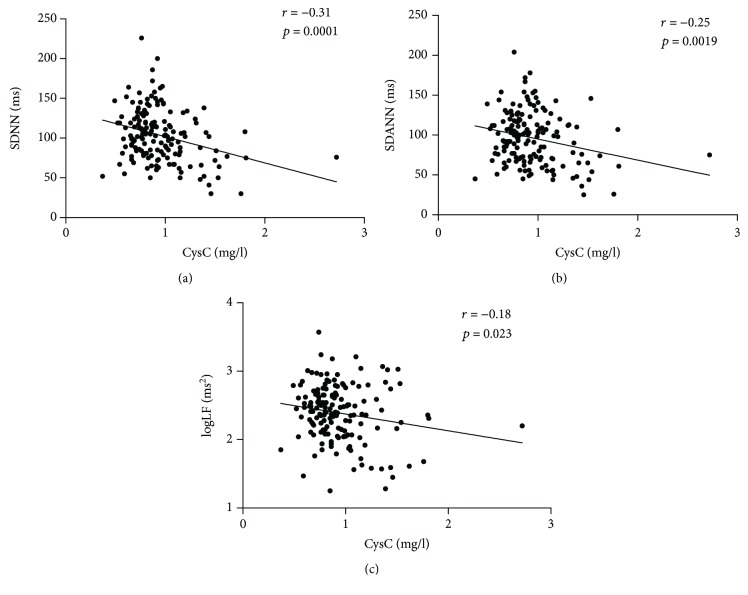
Relationship between cystatin C (mg/l) and HRV indexes in all subjects. Correlation assessed by Pearson analysis. (a) Cystatin C (mg/l) with SDNN (ms), (b) cystatin C (mg/l) with SDANN (ms), and (c) cystatin C (mg/l) with logLF (ms^2^).

**Figure 3:**
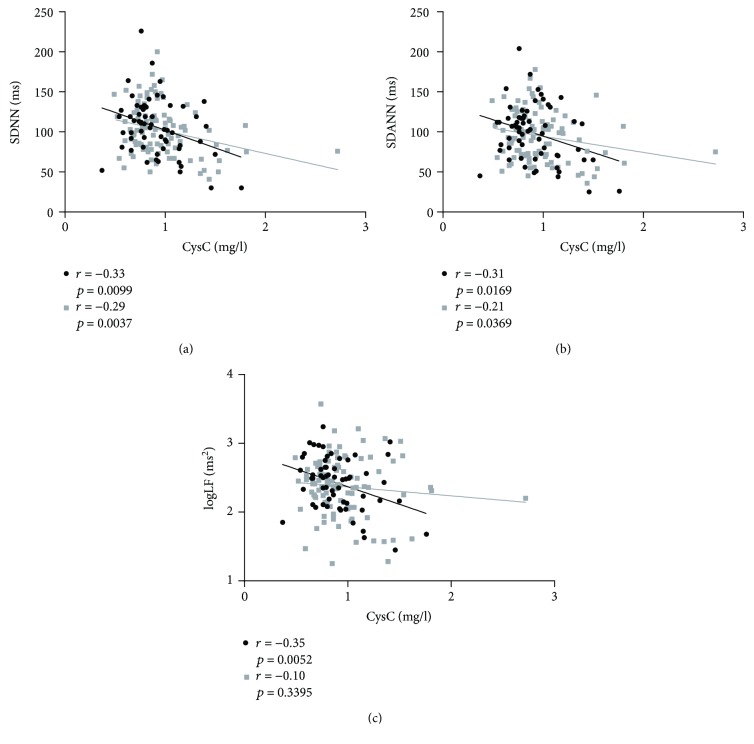
Relationship between cystatin C (mg/l) and HRV indexes in men (black circle) and women (gray square). Correlation assessed by Pearson analysis. (a) Cystatin C (mg/l) with SDNN (ms), (b) cystatin C (mg/l) with SDANN (ms), and (c) cystatin C (mg/l) with logLF (ms^2^).

**Figure 4:**
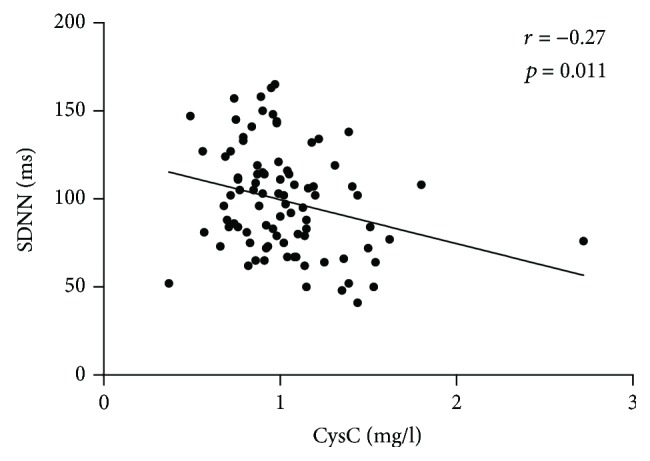
Relationship between cystatin C (mg/l) and SDNN (ms) in subjects over 60 y. Correlation assessed by Pearson analysis.

Correlations between the serum CysC level and other measurements are presented in Table 3 for all subjects. The serum CysC level was positively correlated with age (*r* = 0.40, *P* < 0.001), Cr (*r* = 0.49, *P* < 0.001), UA (*r* = 0.36, *P* < 0.001), and *β*2-MG (*r* = 0.68, *P* < 0.001) and negatively correlated with eGFR (*r* = −0.54, *P* < 0.001) and the HRV indexes, including SDNN (*r* = −0.31, *P* < 0.001), SDANN (*r* = −0.25, *P* = 0.002), and logLF (*r* = −0.18, *P* = 0.023) (Figure 2). Furthermore, the negative relations between CysC concentration and SDNN and SDANN were observed in both genders, while the logLF was correlated with serum CysC only in male patients (*r* = −0.35, *P* = 0.005) (Figure 3). In addition, compared with patients younger than 60 years, only patients older than 60 years were found to have a negative correlation between SDNN and CysC (*r* = −0.27, *P* = 0.011) (Figure 4), while associations between CysC and the other HRV indexes could not be observed in patients younger than 60 years or older than 60 years (*P* > 0.05).

Moreover, the independent relation between CysC levels and the HRV indexes was identified by linear regression analysis. After adjusting for sex, age, BMI, eGFR, blood pressure, and HbA1c, serum CysC levels remained independently associated with SDNN (*β* = −24.11, *P* = 0.015) and SDANN (*β* = −19.88, *P* = 0.047) (Table 4, Table 5).

## 4. Discussion

Cardiovascular autonomic dysfunction often developed without obvious symptoms in type 2 diabetic patients, subsequently leading to cardiovascular events and increasing the cardiovascular mortality among patients [3, 18]. Previous studies revealed that the parasympathetic dysfunction, shown by decreased SDNN and SDANN, was the major cause of altered cardiovascular autonomic function [6, 19, 20]. As the impairment of cardiovascular autonomic function has been proved to be reversed with interventions for risk factors in the very early stages [21, 22], the detection of cardiovascular autonomic dysfunction and interventions to reduce the incidence of cardiovascular events in type 2 diabetic patients are of great clinical importance. However, some methods used to detect cardiac autonomic imbalance, such as Ewing tests, heart rate variability (HRV), and cardiac radionuclide imaging, are often restrained in clinical practice for the complicated operation, requirement of patients' good cooperation, and lack of device [7, 23, 24]. To find a sensitive, reliable, and easily conducted method, especially examining some clinical biomarkers, to detect cardiovascular autonomic dysfunction, is urgently needed.

CysC, a small molecular protein, has been proved to be a more sensitive biomarker than Cr for diabetic kidney disease [25, 26]. In line with previous studies, the positive relation with serum Cr and the negative relation with eGFR in our research further demonstrated that the level of CysC may be an indicator of renal function. In recent years, many studies have investigated the association between serum CysC levels and cardiovascular disease [27, 28]. Wang et al. [11] demonstrated that CysC, but not eGFR, was related to the risk of cardiovascular events in patients with mild renal impairment. Similar results were also found among subjects with diabetes, indicating that CysC might be more sensitive for predicting cardiovascular events than eGFR [11, 12]. In addition, the relationship between CysC and diseases of both the central nervous system (CNS) and peripheral nervous system has been reported in several observational studies [29]. In line with these findings, Hu and his group [30] found that the level of serum CysC was increased significantly in type 2 diabetic patients with diabetic peripheral neuropathy (DPN). As shown by increasing numbers of studies, serum CysC is considered predictive of not only kidney function but also the risk of other complications of diabetes, including cardiovascular events and DPN. However, the relationship between serum CysC levels and the presence of cardiovascular autonomic dysfunction in patients with T2DM has remained unclear. As diabetic nephropathy, including elevated Cr, was demonstrated to be a risk factor for cardiovascular diseases and advanced cardiovascular autonomic neuropathy [31] and CysC levels might be superior to Cr or eGFR as an indicator of diabetic kidney disease [32], we hypothesized that CysC could possibly be an early indicator of cardiovascular autonomic dysfunction in diabetic patients even before Cr levels were elevated.

In the present study, our initial assumption was confirmed as we found that HRV indexes reflecting the injury to cardiovascular autonomic nerve function decreased with an increase in serum CysC levels in T2DM patients with normal serum creatinine and eGFR. Moreover, a significant negative relationship between CysC and the HRV indexes was found in the group with the highest level of serum CysC, indicating that a higher CysC concentration was linked to a greater risk of cardiovascular autonomic dysfunction. In addition, after further analysis, a negative correlation was found in patients over 60 years old but not in patients below 60 years old. This finding may suggest that age seems to act as a risk factor for both CysC and cardiac autonomic imbalance. In previous studies, serum CysC concentrations were associated with risk factors such as age, BMI, LDL-C, fasting blood glucose (FBG), and hypertension among patients with type 2 diabetes [33, 34]. These factors were also proved to be associated with the prevalence of cardiovascular autonomic dysfunction [7]. Furthermore, excluding subjects with serum creatinine higher than 133 *μ*mol/l and eGFR lower than 60 ml/min per 1.73 m^2^from our study, in order to eliminate the confounding effect of decreased renal function, helped make our results more convincing.

The HRV analysis, an effective method for the early detection of impaired cardiovascular autonomic function, is more convenient and accurate than other measurements, such as Ewing tests [35, 36]. The SDNN and SDANN components of the HRV indexes are viewed as reflecting vagal activity in both normal and diabetic subjects [17]. In our study, the negative correlation between CysC concentration and SDNN and SDANN may indicate that a higher level of CysC could serve as a predictor of the impairment of cardiac autonomic nerve function, especially cardiac vagus nerve function. While HRV is reliable in the detection of cardiovascular autonomic dysfunction, it is difficult to perform; thus, the association between CysC and the HRV indexes further suggested that CysC could be a reliable biomarker for detecting impaired cardiovascular autonomic function and would be much more convenient than HRV.

Although the mechanism of association between serum CysC and cardiovascular autonomic dysfunction remained unclear, we found that some evidence from previous studies may support our results and explain this phenomenon. First, serum CysC was proved to be a sensitive biomarker for estimating glomerular filtration rate (GFR) in T2DM patients. As demonstrated by a cohort study conducted in Mayo Clinic [37], the severity of diabetic neuropathy was related to diabetic nephropathy. Moreover, several studies [38] have revealed a close association between diabetic nephropathy and an increased risk of cardiovascular events and death. Similarly, in a study by Orlov et al. [39], the relationship between cardiovascular autonomic neuropathy and renal insufficiency has been investigated in patients with type 2 diabetes. Therefore, the close relationship between serum CysC level and cardiovascular autonomic dysfunction may be attributed to the association between diabetic nephropathy and diabetic cardiovascular autonomic dysfunction. Second, the critical pathogenic elements of cardiovascular autonomic dysfunction, including inflammation, oxidative stress, and endothelial dysfunction, were also related to CysC. Therefore, the close association between CysC and cardiovascular autonomic dysfunction might be explained by the common pathways involved, and further studies should be conducted to explore the effect of serum CysC levels in cardiovascular autonomic dysfunction.

There were some limitations in our study. First, the sample size in our study was relatively small. Second, for better exploring factors in relation to the impairment of cardiovascular autonomic function, the patients enrolled in our study were all inpatients, which were more likely to be identified of cardiovascular autonomic dysfunction than outpatients. As a result, the conclusion cannot be extrapolated to outpatients. Finally, as this study used a cross-sectional design, the results in our study could demonstrate only the relation between CysC and depressed cardiovascular autonomic function rather than the cause and effect. However, based on our results, we still believe that our results could provide evidence that CysC is a reliable and easily examined biomarker of cardiovascular autonomic dysfunction. Prospectively designed studies with larger cohorts including both inpatients and outpatients will be needed in future investigations.

## 5. Conclusions

In conclusion, higher serum CysC was associated with impaired cardiovascular autonomic function, especially the injury to parasympathetic nerve function in type 2 diabetic patients. CysC might be a reliable and convenient biomarker for detecting cardiovascular autonomic dysfunction.

## Figures and Tables

**Table 1:** Characteristics of patients with type 2 diabetes according to serum cystatin C tertiles.

	Tertile 1 (<0.78)	Tertile 2 (0.78–0.99)	Tertile 3 (>0.99)	Total	*P* value		
					Tertile 1 vs. 2	Tertile 1 vs. 3	Tertile 2 vs. 3
*n*	52	57	52	161			
Men (%)	21 (40)	22 (29)	18 (35)	61 (38)			
Age (years)	52.3 ± 10.7	60.3 ± 10.9	66.3 ± 12.1	59.7 ± 12.6	<0.01	<0.01	<0.01
Diabetes duration (years)	5.7 ± 1.3	7.8 ± 2.1	11.1 ± 3.3	8.2 ± 2.5	0.08	<0.01	<0.01
BMI (kg/m^2^)	25.01 ± 3.4	23.82 ± 3.0	24.35 ± 2.9	24.44 ± 3.1	0.07	0.24	0.53
SBP (mmHg)	135.4 ± 17.3	136.3 ± 20.0	135.7 ± 20.3	135.8 ± 19.2	0.96	0.96	0.91
DBP (mmHg)	81.5 ± 10.5	78.8 ± 10.4	76.3 ± 14.8	78.3 ± 12.2	0.08	0.01	0.39
HbA1c (%)	8.4 ± 2.1	8.2 ± 2.3	7.8 ± 2.1	8.2 ± 2.2	0.36	0.11	0.47
TC (mmol/l)	4.88 ± 1.3	4.81 ± 1.1	4.66 ± 1.5	4.78 ± 1.3	0.80	0.40	0.54
TG (mmol/l)	1.63 ± 1.2	1.65 ± 1.6	1.30 ± 0.6	1.53 ± 1.2	0.93	0.17	0.13
HDL-C (mmol/l)	1.20 ± 0.3	1.14 ± 0.3	1.34 ± 1.0	1.23 ± 0.7	0.66	0.30	0.13
LDL-C (mmol/l)	3.07 ± 0.9	3.05 ± 0.9	2.91 ± 1.4	3.01 ± 1.1	0.89	0.45	0.51
B2-MG (*μ*g/l)	1.65 ± 0.4	2.02 ± 0.5	3.01 ± 0.9	2.23 ± 0.8	0.22	0.69	0.42
Cr (*μ*mol/l)	62.71 ± 13.7	68.65 ± 12.2	86.88 ± 23.4	72.62 ± 19.8	0.07	<0.01	<0.01
eGFR (ml/min/1.73 m^2^)	109.57 ± 22.23	95.15 ± 18.90	73.58 ± 22.55	92.84 ± 25.65	<0.01	<0.01	<0.01
FBG (mmol/l)	9.23 ± 4.0	8.45 ± 4.3	7.43 ± 3.4	8.34 ± 4.0	0.33	0.02	0.18
UA (*μ*mol/l)	325.50 ± 91.2	339.00 ± 89.6	415.04 ± 119.1	358.92 ± 107.1	0.48	<0.01	<0.01
SDNN (ms)	110.10 ± 30.6	114.02 ± 34.7	86.37 ± 26.8	103.83 ± 33.1	0.51	<0.01	<0.01
SDANN (ms)	100.75 ± 28.9	104.32 ± 34.3	83.76 ± 31.5	96.53 ± 32.8	0.56	<0.01	<0.01
LogRMSSD (ms)	1.39 ± 0.18	1.43 ± 0.21	1.39 ± 0.24	1.40 ± 0.21	0.40	0.92	0.45
LogPNN50 (%)	0.77 ± 0.43	0.71 ± 0.52	0.64 ± 0.56	0.71 ± 0.51	0.55	0.25	0.55
LogLF (ms^2^)	2.46 ± 0.34	2.42 ± 0.36	2.27 ± 0.48	2.38 ± 0.40	0.60	0.02	0.05
LogHF (ms^2^)	2.10 ± 0.38	2.07 ± 0.4	1.93 ± 0.43	2.03 ± 0.41	0.82	0.05	0.07
LogLF/HF	0.39 ± 0.19	0.34 ± 0.26	0.32 ± 0.35	0.35 ± 0.27	0.42	0.19	0.60
CysC (mg/l)	0.68 ± 0.09	0.88 ± 0.06	1.27 ± 0.3	0.94 ± 0.3			

BMI: body mass index; SBP: systolic blood pressure; DBP: diastolic blood pressure; HbA1c: hemoglobin A1c; TC: total cholesterol; TG: triglyceride; HDL-C: HDL-cholesterol; LDL-C: LDL-cholesterol; Cr: creatinine; eGFR: estimated glomerular filtration rate; FBG: fasting blood glucose; UA: uric acid; *β*2-MG: *β*2-microglobulin; CysC: cystatin C.

**Table 2:** The values of CysC and the HRV indexes in patients of different ages.

	<60 years (*n* = 72)	≥60 years (*n* = 89)	*P* value
CysC (mg/l)	0.84 ± 0.24	1.02 ± 0.32	<0.001
SDNN (ms)	109.90 ± 36.05	99.00 ± 29.95	0.044
SDANN (ms)	101.17 ± 34.61	92.84 ± 30.91	>0.05
RMSSD (ms)	27.29 ± 12.33	30.01 ± 19.29	>0.05
LogPNN50 (%)	0.71 ± 0.46	0.71 ± 0.55	>0.05
LogLF (ms^2^)	2.42 ± 0.41	2.36 ± 0.42	>0.05
LogHF (ms^2^)	2.08 ± 0.41	2.01 ± 0.44	>0.05
LogLF/HF	0.34 ± 0.24	0.35 ± 0.29	>0.05

CysC: cystatin C.

**Table 3:** Relationship between CysC and other measurements in all patients.

	CysC (mg/l)	
	*r*	*P* value
*β*2-MG (*μ*g/l)	0.68	<0.001
UA (*μ*mol/l)	0.36	<0.001
HbA1C (%)	-0.95	0.234
Age (years)	0.40	<0.001
Cr (*μ*mol/l)	0.49	<0.001
eGFR (ml/min/1.73 m^2^)	-0.54	< 0.001
BMI (kg/m^2^)	-0.43	0.587
SDNN (ms)	-0.31	<0.001
SDANN (ms)	-0.25	0.002
LogLF (ms^2^)	-0.18	0.023
LogHF (ms^2^)	-0.09	0.272
LogRMMSD (ms)	-0.053	0.504
LogPNN50 (%)	0.013	0.884
LogLF/HF	-0.14	0.088

*β*2-MG: *β*2-microglobulin; CysC: cystatin C; UA: uric acid; HbA1c: hemoglobin A1c; Cr: creatinine; eGFR: estimated glomerular filtration rate; BMI: body mass index.

**Table 4:** Linear regression analysis of SDNN with different clinical characteristics.

	Model 1		Model 2		Model 3	
	*β*	*P* value	*β*	*P* value	*β*	*P* value
CysC (mg/l)	-33.05	<0.001	-26.35	0.005	-24.11	0.015
Age (years)			-0.37	0.120	-0.67	0.010
Gender			-0.01	0.116	-1.45	0.781
BMI (kg/m^2^)			-1.04	0.203	-1.03	0.203
HbA1c (%)					-4.07	0.001
eGFR (ml/min/1.73 m^2^)					0.08	0.522
Systolic BP (mmHg)					0.30	0.071
Diastolic BP (mmHg)					-0.50	0.073

BMI: body mass index; BP: blood pressure; eGFR: estimated glomerular filtration rate. Model 1: unadjusted model. Model 2: adjusted by age, gender, and BMI. Model 3: adjusted by model 2 plus HbA1c, eGFR, systolic BP, and diastolic BP.

**Table 5:** Linear regression analysis of SDANN with different clinical characteristics.

	Model 1		Model 2		Model 3	
	*β*	*P* value	*β*	*P* value	*β*	*P* value
CysC (mg/l)	-26.37	<0.001	-20.32	0.005	-19.88	0.047
Age (years)			-0.30	0.198	-0.65	0.012
Gender			0.13	0.982	-1.53	0.773
BMI (kg/m^2^)			-0.74	0.373	-0.70	0.391
HbA1C (%)					-3.74	0.003
eGFR (ml/min/1.73 m^2^)					0.02	0.881
Systolic BP (mmHg)					0.31	0.060
Diastolic BP (mmHg)					-0.58	0.040

BMI: body mass index; BP: blood pressure; eGFR: estimated glomerular filtration rate. Model 1: unadjusted model. Model 2: adjusted by age, gender, and BMI. Model 3: adjusted by model 2 plus HbA1c, eGFR, systolic BP, and diastolic BP.

## Data Availability

We confirm that the data in our study are available from the corresponding author, Dr Bin Yao, upon request.
